# Factors affecting psychological health and career choice among medical students in eastern and western region of China after COVID-19 pandemic

**DOI:** 10.3389/fpubh.2023.1081360

**Published:** 2023-03-08

**Authors:** Jingxian Wang, Chunhua Yang, Jingzhen Wang, Xingling Sui, Wen Sun, Yue Wang

**Affiliations:** ^1^School of Clinical and Basic Medical Sciences, Shandong Provincial Hospital Affiliated to Shandong First Medical University, Jinan, China; ^2^Department of Statistics, Institute of Shandong Evidence-Based Medicine, Jinan, China; ^3^Queen Mary School, Nanchang University, Nanchang, China; ^4^Medical Science and Technology Innovation Center, Shandong First Medical University and Shandong Academy of Medical Sciences, Jinan, China

**Keywords:** post-epidemic era, medical students, career choice, different regions in China, psychological health

## Abstract

**Introduction:**

To unearth superior countermeasures that improve psychological health and upgrade the quality of employment for medical students in China in post-epidemic era, this study was designed to determine the possible factors affecting psychological status and future career choice of this population.

**Methods:**

A cross-sectional observational study was carried out. Depression Anxiety Stress Scale-21 (DASS-21) and Insomnia Severity Index (ISI) were applied to measure psychological state. Chi-square and logistic regression analyses were adopted to filtrate related factors for psychological health and employment intention.

**Results:**

A total of 936 medical students, including 522 from eastern universities and 414 from western universities, were enrolled in the study. Anxiety among students in China's western universities was higher than that in China's eastern universities (30.4% vs. 22.0%), but no differences in the occurrences of stress (11.4% vs. 13.4%), depression (28.7% vs. 24.5%) and insomnia (30.7% vs. 25.7%). Grades, academic ranking, household income, attitudes about COVID-19 were associated with the occurrence of psychological problems. In addition, major, education level, academic ranking, family income, and clinical experience may affect the choice of future employment location and employment income. Notably, household income affected by COVID-19 and the perception of epidemic prevention and control resulted in changes in future employment region and income. COVID-19 can lead medical students with psychological problems to have a negative attitude toward future employment. Encouragingly, multiple activities, namely, proactive consideration of employment, taking part in career planning training lectures and timely adjustment of career planning, were beneficial to the professional identity of medical students.

**Conclusion:**

This study suggests that medical student psychology is influenced by COVID-19 and academic and financial pressures; actively coping with COVID-19 and making career planning in advance will contribute to optimizing future employment. Our findings provide a potent guideline for relevant departments to accurately adjust job deployment and for medical students to actively choose a career in the future.

## Introduction

Faced with the sudden coronavirus disease (COVID-19) epidemic in 2019, medical students who have been already burdened with strong employment pressure are now facing more severe challenges and have greater psychological problems (1). Since June 2020, the COVID-19 epidemic in China has been effectively controlled and has entered the post-epidemic era (2). Although the normal order of social life and production is gradually being restored, there are still many uncertainties about future employment prospects, and the prevention and control of psychological health problems or challenges are new.

Multiple studies have shown that medical students' psychological health and occupational planning have been affected to varying degrees by the pandemic (3–5). According to a cross-sectional study of medical students during the early outbreak in February 2020, male students and students focusing less on COVID-19 have a high risk of depressive symptoms, while students in fifth grade and students with high concerns about the negative impact of COVID-19 on their education or employment are prone to have poor sleep quality (6). Another survey has demonstrated that more than one-third of medical students suffer from depression during this outbreak (7). Medical and health care students are ascertained to affected by the pandemic in their choices of majors, workplaces and employment duration (8). Additionally, in accordance with the survey results of Wang et al., approximately a quarter of medical students have suffered some degree of anxiety and 13% experienced some degree of depression during the severe respiratory syndrome coronavirus 2 (SARS-CoV-2) pandemic, and approximately 4% wanted to give up engaging in primary medical care (5). Since the outbreak of the epidemic, doctors and nurses have been the front-line of epidemic prevention and control. As the future successors of the country's medical and health reserve undertake the fight against the epidemic, medical students' psychological problems, career choices, and professional identity are particularly important for societal development in the post-epidemic era. Therefore, it is necessary to further identify related factors that may affect psychological disorders and career planning of medical students as soon as possible. At present, there is still a lack of investigations into the comprehensive factors affecting the psychological health and career planning of medical students under the background of the post-epidemic era. For this purpose, a cross-sectional survey was conducted among medical students in China's eastern and western regions in this study. The findings can provide relatively comprehensive guidance for the employment of medical students, and can be helpful for colleges and related departments in adjusting their work deployment conditions.

## Materials and methods

### Participants, questionnaire formation and data collection

A cross-sectional study was performed using an online survey, Questionnaire Star (https://www.wjx.cn), running from June 19 to July 2, 2022 and our data source was published by this platform. The questionnaire consisted of five parts: general information, including location of universities, gender, major, grade, score ranking, degree of education, marital status, home place, average monthly household income, whether participant had an organic disease; attitudes toward COVID-19; psychological indicators; internship training experience; and career planning mentality for this epidemic. The inclusion criteria were used: medical students who fully understood the purpose and significance of the study and were able to cooperate with the researchers in conducting research activities. The exclusion criteria were used: students who already had a psychiatric disorder prior to COVID-19 outbreak or were under observation due to suspected or confirmed COVID-19 were excluded from this study. This study was approved by the Ethic Committee of Shandong First Medical University. All respondents were informed in advance that participation was anonymous and voluntary. Consent was denoted if the participants began to answer the questionnaire.

### Psychological indicators

The Depression Anxiety and Stress Scale-21 (DASS-21) (9) and Insomnia Severity Index (ISI) (10) were used to evaluate outcomes of depressive/anxious/stressful symptoms and insomnia symptoms by discrimination of 21 items and 7 items, respectively. The grading standards are listed in Tables 1, 2 according to the subscale scores.

**Table 1:** DASS-21 subscale classification criteria.

**Degree level**	**Depression**	**Anxiety**	**Stress**
Normal	0–9	0–7	0–14
Mild	10–13	8–9	15–18
Moderate	14–20	10–14	19–25
Severe	21–27	15–19	26–33
Extreme	≥28	≥20	≥34

**Table 2:** Insomnia severity index scale.

**Degree level**	**Total points (range 0–28)**
No clinically significant insomnia	0–7
Subthreshold insomnia	8–14
Clinical insomnia (moderate to severe)	15–21
Clinical insomnia (severe)	22–28

### Statistical analysis

SPSS version 22.0 was used to analyze all data. Chi-square with Fisher's exact test was applied to compare differences in categorical variables between groups. Binary or multiple logistic regression analysis was adopted to distinguish the risk and protective factors for psychological health and employment intention. A *p* value < 0.05 was considered statistically significant.

## Results

### The general profile of medical students

In this study, 1,099 questionnaires were received from students in several comprehensive or medical universities in eastern and western China, among which 1,004 were from medical and closely related majors. After excluding unqualified questionnaires according to the exclusion criteria, a total of 936 questionnaires were valid. General characteristics of these 936 medical students were shown in Table 3. 522 (55.8%) were from China's eastern universities. Majority were female (69.4%), and most majors were nursing (39.6%) or clinical medicine (36.9%). Freshmen (50.2%), sophomores (34.5%) and juniors (10.4%) make up the majority of grades. Students were categorized by academic rankings: top 25% (26.0), 25%−50% (37.3%), 50%−75% (26.9%), and below 75% (9.8%). Participants' education level varied from junior college (15.7%) and undergraduate degrees (76.7%) to graduate degrees (7.6%). The vast majority were unmarried (99.7%) and no organic disease (98.5%). They were born in metropolises (9%), small-medium cities (49.4%) and countryside (41.7%). Most participants had an average monthly household income < 10,000 yuan (83.4%).

**Table 3:** Distribution of descriptive characteristics of respondents.

**Item category**	**Classification**	**Population (*n*/%)**	**Item category**	**Classification**	**Population (*n*/%)**
Location of universities	Eastern	522 (55.8)	Degree of education	Junior college	147 (15.7)
	Western	414 (44.2)		Undergraduate	718 (76.7)
Gender	Male	286 (30.6)		Graduate	71 (7.6)
	Female	650 (69.4)	Marital status	Married	3 (0.3)
Major	Nursing	371 (39.6)		Unmarried	933 (99.7)
	Clinical medicine	345 (36.9)	Home place	Metropolis	84 (9.0)
	Others	220 (23.5)		Small-medium cities	462 (49.4)
Grade	Freshman	470 (50.2)		Countryside	390 (41.7)
	Sophomore	323 (34.5)	Average monthly household income	< 5,000	403 (43.1)
	Junior	97 (10.4)		5,000–10,000	378 (40.4)
	Senior and intern	37 (4.0)		>10,000	155 (16.6)
	Master/PhD graduate student	9 (1.0)	Whether have organic disease	Yes	14 (1.5)
Score ranking	The top 25%	243 (26.0)		No	922 (98.5)
	25%−50%	349 (37.3)			
	50%−75%	252 (26.9)			
	Below 75%	92 (9.8)			

### Attitudes about COVID-19 and their association with psychological indicators

In terms of attitudes toward outbreak, Table 4 exhibits that participants were very concerned (27.5%), moderately concerned (39%), somewhat (27.7%), slightly concerned (4.9%) and unconcerned (1%). Most household incomes were affected by the pandemic (67.2%). Half of them participated in epidemic prevention and control work (51.4%). Most participants had no friends or relatives infected (98.1%) and did not mind others infected with COVID-19 (90.5%).

**Table 4:** Cognition and behavior of medical students on the pandemic.

**Item**	**Statistical index**	**Population (*n*/%)**
Are you still following the news of the pandemic?	Very concerned, willing to actively search the recent situation	257 (27.5)
	More concerned, will search for interested topics	365 (39.0)
	Medium, read about it, but don't actively search for it	259 (27.7)
	Don't pay much attention, understand the general situation, but don't care about the specific data	46 (4.9)
	Unconcerned and uninterested in news of the epidemic	9 (1.0)
Have household incomes been affected by the pandemic?	Yes	629 (67.2)
	No	307 (32.8)
Have you participated in pandemic prevention and control work?	Yes	481 (51.4)
	No	455 (48.6)
Have any friends or relatives been infected with COVID-19?	Yes	18 (1.9)
	No	918 (98.1)
Do you mind students who have been infected with COVID-19?	Yes	89 (9.5)
	No	847 (90.5)

**Figure 1:**
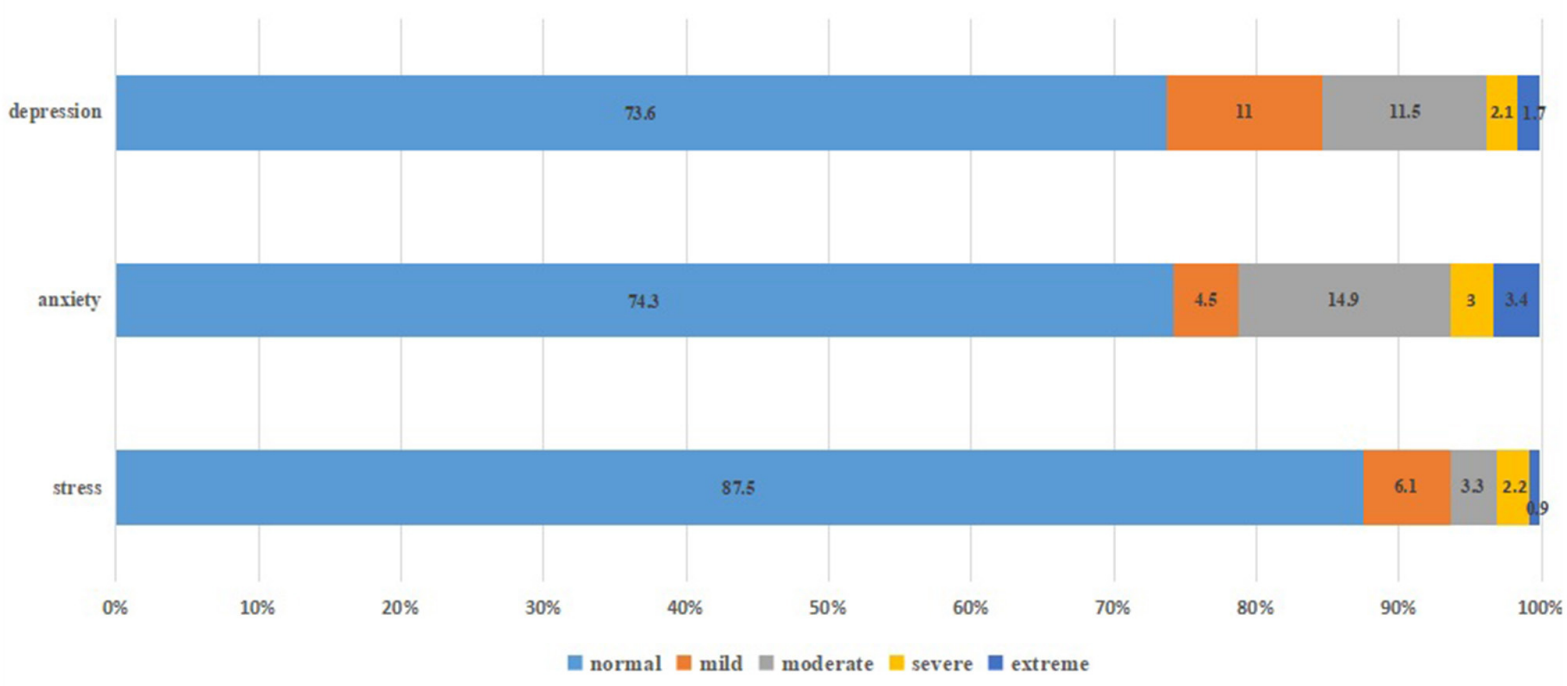
Overall distribution of DASS-21 and insomnia severity index.

**Figure 2:**
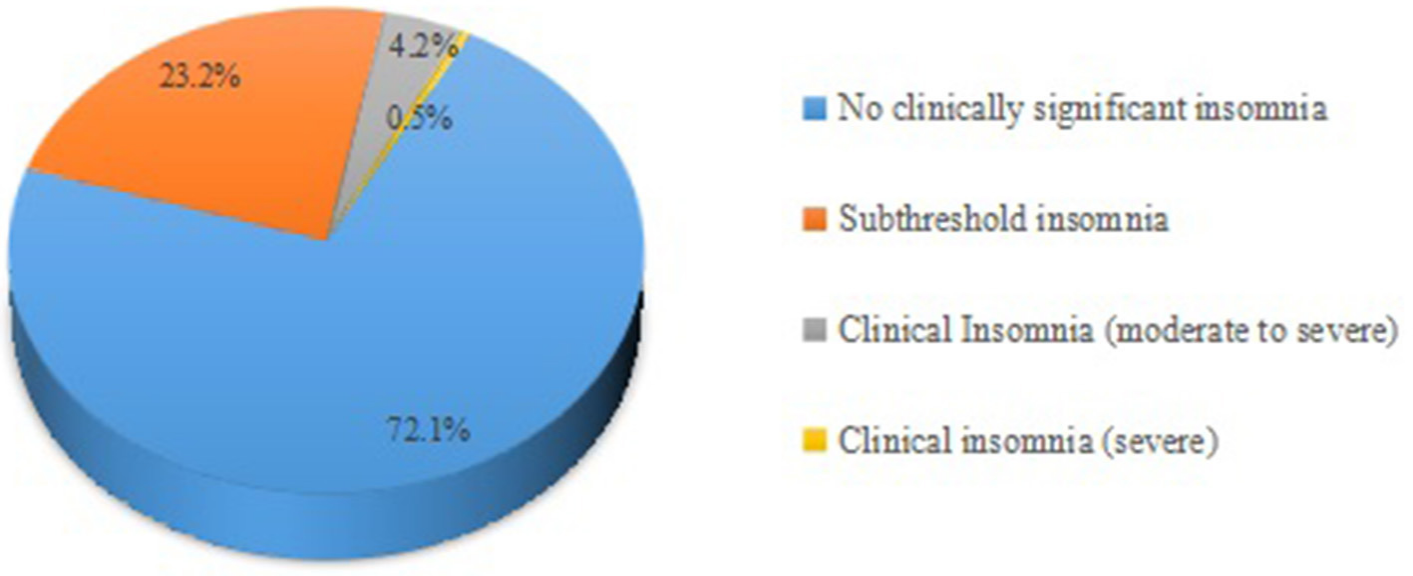
Overall distribution of insomnia severity index scale.

Figures 1, 2 showed that most students' mental health was normal, but they still had psychological symptoms, instantiating stress (12.5%), anxiety (25.7%), depression (26.4%), and insomnia (27.9%). As shown in Table 5, medical students in China's western universities were more likely to report anxiety than those in China's eastern universities (*p* = 0.003), but not stress, depression and insomnia (All *p* > 0.05). There were differences in occurrences of stress, anxiety and insomnia among medical students with different majors (*p* < 0.001, *p* = 0.002 and *p* = 0.001, respectively). Grade level affected anxiety (*p* = 0.001), and score rankings affected depressive symptoms (*p* = 0.006): the lower the academic ranking was, the more likely depression symptoms were to appear. Household income related with anxiety (*p* = 0.008), and students from middle-income families were less likely to suffer from psychological problems. Moreover, attitudes toward COVID-19 have been suggested to be related to their psychological conditions. For instance, having not worked in COVID-19 prevention and control (*p* = 0.008) and their close friends or family members infected with COVID-19 increased the possibilities of experiencing anxiety and depression (*p* = 0.027, *p* = 0.011, respectively). Students who cared that others had been infected were at increased risk of stress and insomnia (*p* = 0.001, *p* = 0.042, respectively).

**Table 5:** Analysis of the factors affecting psychological health of the participants.

**Category**	**Variable**	**Classification**	**Total number**	**Stress**			**Anxiety**			**Depression**			**Insomnia**		
				**Normal population (%)**	**Symptomatic population (%)**	***p* value**	**Normal population (%)**	**Symptomatic population (%)**	***p* value**	**Normal population (%)**	**Symptomatic population (%)**	***p* value**	**Normal population (%)**	**Symptomatic population (%)**	***p* value**
General information	Location of university	Eastern	522	452 (86.6)	70 (13.4)	0.345	407 (78.0)	115 (22.0)	0.003	394 (75.5)	128 (24.5)	0.145	388 (74.3)	134 (25.7)	0.090
		Western	414	367 (88.6)	47 (11.4)		288 (69.6)	126 (30.4)		295 (71.3)	119 (28.7)		287 (69.3)	127 (30.7)	
	Gender	Male	286	244 (85.3)	42 (14.7)	0.180	211 (73.8)	75 (26.2)	0.825	197 (68.9)	89 (31.1)	0.029	201 (70.3)	85 (29.7)	0.406
		Female	650	575 (88.5)	75 (11.5)		484 (74.5)	166 (25.5)		492 (75.7)	158 (24.3)		474 (72.9)	176 (27.1)	
	Major	Nursing	371	326 (87.9)	45 (12.1)	0.000	259 (69.8)	112 (30.2)	0.002	267 (72.0)	104 (28.0)	0.108	255 (68.7)	116 (31.3)	0.001
		Clinical medicine	345	284 (82.3)	61 (17.7)		254 (73.6)	91 (26.4)		248 (71.9)	97 (28.1)		240 (69.6)	105 (30.4)	
		Others	220	209 (95.0)	11 (5.0)		182 (82.7)	38 (17.3)		174 (79.1)	46 (20.9)		180 (81.8)	40 (18.2)	
	Degree of education	Junior college	147	134 (91.2)	13 (8.8)	0.210	100 (68.0)	47 (32.0)	0.169	109 (74.1)	38 (25.9)	0.721	105 (71.4)	42 (28.6)	0.577
		Undergraduate	718	626 (87.2)	92 (12.8)		541 (75.3)	177 (24.7)		525 (73.1)	193 (26.9)		515 (71.7)	203 (28.3)	
		Graduate	71	59 (83.1)	12 (16.9)		54 (76.1)	17 (23.9)		55 (77.5)	16 (22.5)		55 (77.5)	16 (22.5)	
	Grade	Freshman	470	416 (88.5)	54 (11.5)	0.293	366 (77.9)	104 (22.1)	0.001	361 (76.8)	109 (23.2)	0.057	353 (75.1)	117 (24.9)	0.176
		Sophomore	323	281 (87.0)	42 (13.0)		220 (68.1)	103 (31.9)		224 (69.3)	99 (30.7)		220 (68.1)	103 (31.9)	
		Junior	97	79 (81.4)	18 (18.6)		67 (69.1)	30 (30.9)		66 (68.0)	31 (32.0)		66 (68.0)	31 (32.0)	
		Senior and intern	37	34 (91.9)	3 (8.1)		34 (91.9)	3 (8.1)		30 (81.1)	7 (18.9)		29 (78.4)	8 (21.6)	
		Master/PhD graduate student	9	9 (100.0)	0 (0.0)		8 (88.9)	1 (11.1)		8 (88.9)	1 (11.1)		7 (77.8)	2 (22.2)	
	Score ranking	Top 25%	243	215 (88.5)	28 (11.5)	0.680	192 (79.0)	51 (21.0)	0.213	198 (81.5)	45 (18.5)	0.006	188 (77.4)	55 (22.6)	0.067
		25%−50%	349	307 (88.0)	42 (12.0)		251 (71.9)	98 (28.1)		253 (72.5)	96 (27.5)		253 (72.5)	96 (27.5)	
		50%−75%	252	220 (87.3)	32 (12.7)		187 (74.2)	65 (25.8)		178 (70.6)	74 (29.4)		175 (69.4)	77 (30.6)	
		Below 75%	92	77 (83.7)	15 (16.3)		65 (70.7)	27 (29.3)		60 (65.2)	32 (34.8)		59 (64.1)	33 (35.9)	
	Marital status	Married	3	3 (100.0)	0 (0.0)	1.000	3 (100.0)	0 (0.0)	0.573	3 (100.0)	0 (0.0)	0.570	2 (66.7)	1 (33.3)	1.000
		Unmarried	933	816 (87.5)	117 (12.5)		692 (74.2)	241 (25.8)		686 (73.5)	247 (26.5)		673 (72.1)	260 (27.9)	
	Home place	Metropolis	84	71 (84.5)	13 (15.5)	0.536	61 (72.6)	23 (27.4)	0.407	65 (77.4)	19 (22.6)	0.137	61 (72.6)	23 (27.4)	0.134
		Small-medium cities	462	409 (88.5)	53 (11.5)		352 (76.2)	110 (23.8)		350 (75.8)	112 (24.2)		346 (74.9)	116 (25.1)	
		Countryside	390	339 (86.9)	51 (13.1)		282 (72.3)	108 (27.7)		274 (70.3)	116 (29.7)		268 (68.7)	122 (31.3)	
	Average monthly household income	< 5,000	403	353 (87.6)	50 (12.4)	0.986	284 (70.5)	119 (29.5)	0.008	287 (71.2)	116 (28.8)	0.323	285 (70.7)	118 (29.3)	0.622
		5,000–10,000	378	331 (87.6)	47 (12.4)		301 (79.6)	77 (20.4)		287 (75.9)	91 (24.1)		279 (73.8)	99 (26.2)	
		>10,000	155	135 (87.1)	20 (12.9)		110 (71.0)	45 (29.0)		115 (74.2)	40 (25.8)		111 (71.6)	44 (28.4)	
	Whether have organic diseases	Yes	14	12 (85.7)	2 (14.3)	0.691	11 (78.6)	3 (21.4)	1.000	11 (78.6)	3 (21.4)	1.000	10 (71.4)	4 (28.6)	1.000
		No	922	807 (87.5)	115 (12.5)		684 (74.2)	238 (25.8)		678 (73.5)	244 (26.5)		665 (72.1)	257 (27.9)	
Knowledge about the pandemic	Have household incomes been affected by the pandemic?	Yes	629	552 (87.8)	77 (12.2)	0.732	459 (73.0)	170 (27.0)	0.200	451 (71.7)	178 (28.3)	0.058	444 (70.6)	185 (29.4)	0.136
		No	307	267 (87.0)	40 (13.0)		236 (76.9)	71 (23.1)		238 (77.5)	69 (22.5)		231 (75.2)	76 (24.8)	
	Have you participated in epidemic prevention and control work?	Yes	481	429 (89.2)	52 (10.8)	0.108	375 (78.0)	106 (22.0)	0.008	373 (77.5)	108 (22.5)	0.005	354 (73.6)	127 (26.4)	0.299
		No	455	390 (85.7)	65 (14.3)		320 (70.3)	135 (29.7)		316 (69.5)	139 (30.5)		321 (70.5)	134 (29.5)	
	Have any friends or relatives been infected with COVID-19?	Yes	18	14 (77.8)	4 (22.2)	0.266	9 (50.0)	9 (50.0)	0.027	8 (44.4)	10 (55.6)	0.011	11 (61.1)	7 (38.9)	0.293
		No	918	805 (87.7)	113 (12.3)		689 (74.7)	232 (25.3)		681 (74.2)	237 (25.8)		664 (72.3)	254 (27.7)	
	Do you mind students who have been infected with COVID-19?	Yes	89	68 (76.4)	21 (23.6)	0.001	62 (69.7)	27 (30.3)	0.298	61 (68.5)	28 (31.5)	0.254	56 (62.9)	33 (37.1)	0.042
		No	847	751 (88.7)	96 (11.3)		633 (74.7)	214 (25.3)		628 (74.1)	219 (25.9)		619 (73.1)	228 (26.9)	

### Discerning relevant factors influencing the choice of future employment region and monthly income in the post-pandemic era

Multiple logistic regression analyses (Table 6) showed that nursing students (*p* = 0.011) and freshmen (*p* = 0.002) tended to choose first-tier cities for employment. Medical students whose family monthly income was < 5,000 yuan tended to choose places near hometowns or places of study (*p* = 0.036). Moreover, medical students whose family incomes were affected by the pandemic were not inclined to choose first-tier cities (*p* = 0.042).

**Table 6:** Analysis of the factors affecting the preferred employment region.

**Category**	**Variable**	**Classification**	**Places near their hometown or their place of study vs. first-tier cities**			**Other cities vs. first-tier cities**		
			***p*** **-value**	**OR**	**95%CI**	***p*** **-value**	**OR**	**95%CI**
General information	Location of universities	Eastern	0.481	0.773	0.377–1.584	0.662	1.336	0.364–4.905
		Western	Referent			Referent		
	Gender	Male	0.669	1.077	0.767–1.513	0.983	1.007	0.559–1.812
		Female	Referent			Referent		
	Major	Nursing	0.011	0.401	0.199–0.808	0.806	0.851	0.236–3.077
		Clinical medicine	0.068	0.665	0.429–1.031	0.985	1.008	0.456–2.225
		Others	Referent			Referent		
	Degree of education	Junior college	0.838	1.082	0.507–2.308	0.056	4.070	0.966–17.152
		Undergraduate	0.612	1.155	0.662–2.014	0.191	2.172	0.679–6.951
		Graduate	Referent			Referent		
	Grade	Freshman	0.002	0.478	0.301–0.759	0.261	0.630	0.281–1.412
		sophomore	0.408	0.811	0.494–1.332	0.931	1.038	0.446–2.417
		Junior and above	Referent			Referent		
	Score ranking	Top 25%	0.716	0.904	0.523–1.561	0.369	0.654	0.259–1.653
		25%−50%	0.503	1.194	0.710–2.009	0.975	1.014	0.432–2.376
		50%−75%	0.285	1.339	0.784–2.287	0.416	1.427	0.606–3.362
		Below 75%	Referent			Referent		
	Home place	Metropolis	0.086	0.611	0.348–1.071	0.167	0.434	0.133–1.417
		Small-medium cities	0.495	1.122	0.806–1.563	0.511	1.204	0.692–2.094
		Countryside	Referent			Referent		
	Average monthly household income	< 5,000	0.036	1.693	1.036–2.767	0.335	1.513	0.652–3.515
		5,000–10,000	0.375	1.222	0.785–1.901	0.972	1.014	0.463–2.224
		>10,000	Referent			Referent		
	Whether have organic disease	Yes	0.958	0.966	0.269–3.469	0.303	2.283	0.475–10.960
		No	Referent			Referent		
Psychological factors	Stress	Yes	0.220	1.397	0.818–2.386	0.512	0.751	0.320–1.766
		No	Referent			Referent		
	Anxiety	Yes	0.972	0.992	0.615–1.599	0.652	1.209	0.530–2.758
		No	Referent			Referent		
	Depression	Yes	0.576	0.872	0.540–1.409	0.567	0.791	0.355–1.764
		No	Referent			Referent		
	Insomnia	Yes	0.603	1.102	0.764–1.592	0.489	1.243	0.671–2.301
		No	Referent			Referent		
Internship training experience	Have you thought about what you will do after graduation?	Yes	0.347	1.225	0.802–1.871	0.460	0.789	0.420–1.481
		No	Referent			Referent		
	Have attended any training or lecture related to career orientation	Yes	0.574	0.915	0.670–1.248	0.766	0.921	0.537–1.580
		No	Referent			Referent		
	Clinical practice experience	Yes	0.772	0.943	0.633–1.405	0.055	0.457	0.206–1.016
		No	Referent			Referent		
Knowledge about the pandemic	Are you still following the news of the epidemic?	Very concerned	0.526	1.761	0.306–10.126	0.056	0.152	0.022–1.051
		More concerned	0.367	2.223	0.391–12.633	0.171	0.268	0.041–1.763
		Medium	0.387	2.151	0.379–12.216	0.308	0.379	0.059–2.446
		Don't pay much attention	0.314	2.568	0.409–16.141	0.719	0.692	0.093–5.165
		Unconcerned	Referent			Referent		
	Have household incomes been affected by the pandemic?	Yes	0.130	1.289	0.928–1.791	0.042	1.876	1.024–3.437
		No	Referent			Referent		
	Have you participated in epidemic prevention and control work?	Yes	0.361	0.870	0.645–1.173	0.569	1.163	0.692–1.954
		No	Referent			Referent		
	Have any friends or relatives been infected with COVID-19?	Yes	0.466	1.494	0.508–4.397	0.699	0.645	0.070–5.974
		No	Referent			Referent		
	Do you mind students who have been infected with COVID-19?	Yes	0.112	0.670	0.409–1.098	0.740	1.132	0.545–2.349
		No	Referent			Referent		

As shown in Table 7, nursing (*p* = 0.027), clinical medicine (*p* = 0.026) students and postgraduate students (all *p* < 0.05) were more likely to obtain monthly salaries >8,000 yuan. Students with academic scores in the bottom 50% and from families with an average monthly income < 10,000 yuan may expect their future monthly salaries to be < 8,000 yuan (*p* < 0.05). Students who had clinical internship experience (*p* = 0.003), participated in epidemic prevention work (both *p* < 0.05) and cared that people around them had been infected with COVID-19 (*p* = 0.017) were more likely to obtain their future monthly incomes >15,000 yuan.

**Table 7:** Analysis of the factors affecting expected employment monthly income.

**Category**	**Variable**	**Classification**	**8,000–15,000 yuan vs. 3,000–8,000 yuan**			**Above 15,000 yuan vs. 3,000–8,000 yuan**		
			***p-*** **value**	**OR**	**95%CI**	***p*** **-value**	**OR**	**95%CI**
General information	Location of universities	Eastern	0.077	1.992	0.929–4.274	0.389	1.592	0.553–4.588
		Western	Referent			Referent		
	Gender	Male	0.533	1.128	0.773–1.645	0.269	1.295	0.819–2.048
		Female	Referent			Referent		
	Major	Nursing	0.027	2.330	1.101–1.931	0.352	1.627	0.583–4.537
		Clinical medicine	0.026	1.699	1.066–2.706	0.027	1.982	1.083–3.630
		Others	Referent			Referent		
	Degree of education	Junior college	0.002	0.244	0.098–0.607	0.001	0.137	0.044–0.428
		Undergraduate	0.026	0.439	0.213–0.906	0.040	0.427	0.190–0.961
		Graduate	Referent			Referent		
	Grade	Freshman	0.100	1.519	0.923–2.500	0.199	1.508	0.806–2.820
		Sophomore	0.770	1.084	0.632–1.860	0.111	1.714	0.883–3.327
		Junior and above	Referent			Referent		
	Score ranking	Top 25%	0.063	1.808	0.968–3.379	0.881	0.947	0.467–1.920
		25%−50%	0.455	1.253	0.693–2.267	0.388	0.747	0.385–1.449
		50%−75%	0.645	1.152	0.631–2.102	0.018	0.428	0.211–0.865
		Below 75%	Referent			Referent		
	Home place	Metropolis	0.682	1.149	0.592–2.229	0.411	1.381	0.639–2.986
		Small-medium cities	0.462	0.876	0.616–1.246	0.253	0.757	0.469–1.221
		Countryside	Referent			Referent		
	Average monthly household income	< 5,000	0.000	0.302	0.167–0.546	0.000	0.142	0.073–0.278
		5,000–10,000	0.013	0.495	0.285–0.862	0.000	0.211	0.115–0.388
		>10,000	Referent			Referent		
	Whether have organic disease	Yes	0.802	1.182	0.321–4.355	0.744	1.307	0.262–6.514
		No	Referent			Referent		
Psychological factors	Stress	Yes	0.304	0.727	0.396–1.335	0.152	0.602	0.301–1.205
		No	Referent			Referent		
	Anxiety	Yes	0.694	0.898	0.527–1.531	0.097	0.576	0.301–1.104
		No	Referent			Referent		
	Depression	Yes	0.972	0.991	0.589–1.666	0.740	0.895	0.467–1.718
		No	Referent			Referent		
	Insomnia	Yes	0.554	0.886	0.594–1.322	0.905	0.969	0.582–1.615
		No	Referent			Referent		
Internship training experience	Have you thought about what you will do after graduation?	Yes	0.335	0.800	0.508–1.260	0.727	0.902	0.507–1.607
		No	Referent			Referent		
	Have attended any training or lecture related to career orientation	Yes	0.633	1.085	0.776–1.519	0.089	0.679	0.434–1.060
		No	Referent			Referent		
	Clinical practice experience	Yes	0.167	1.371	0.877–2.143	0.003	2.288	1.319–3.969
		No	Referent			Referent		
Knowledge about the pandemic	Are you still following the news of the epidemic?	Very concerned	0.888	1.167	0.135–10.017	0.725	0.692	0.089–5.387
		More concerned	0.710	1.502	0.175–12.878	0.668	0.640	0.084–4.909
		Medium	0.684	1.563	0.182–13.399	0.609	0.588	0.077–4.499
		Don't pay much attention	0.979	0.970	0.102–9.187	0.558	0.525	0.061–4.536
		Unconcerned	Referent			Referent		
	Have household incomes been affected by the pandemic?	Yes	0.664	1.084	0.754–1.557	0.936	0.981	0.618–1.558
		No	Referent			Referent		
	Have you participated in epidemic prevention and control work?	Yes	0.031	1.428	1.033–1.974	0.037	1.560	1.028–2.367
		No	Referent			Referent		
	Have any friends or relatives been infected with COVID-19?	Yes	0.576	0.668	0.163–2.740	0.229	2.209	0.608–8.031
		No	Referent			Referent		
	Do you mind students who have been infected with COVID-19?	Yes	0.972	0.990	0.560–1.748	0.017	2.139	1.144–4.000
		No	Referent			Referent		

### Changes in professional satisfaction and career planning before and after the pandemic

From Table 8 displaying the distribution of changes in their career plans before/after this pandemic, half of the students expressed the opinion that the job situation was worse now than before the pandemic. More than half of the students thought their satisfactions with their majors and future career plans had not changed significantly than before the pandemic. Above half of the students felt great job pressure and were ready to actively deal with the pressure in the context of the normalization of pandemic. Half of the students would not actively advise others to study medical-related majors.

**Table 8:** Analysis of employment and career planning situation after the pandemic.

**Items**	**Statistical indicators**	**Population (*n*/%)**
What do you think of the current employment situation after this pandemic?	It's worse than before.	482 (51.5)
	It's about the same as before.	201 (21.5)
	More opportunities than before	253 (27.0)
Are you more satisfied with your major now than before?	More satisfactory	300 (32.1)
	There is not much difference	588 (62.8)
	Unsatisfactory	48 (5.1)
Has your career plan changed during this pandemic?	Yes	194 (20.7)
	No	742 (79.3)
Do you feel the pressure of employment in the context of the ongoing pandemic?	Great	287 (30.7)
	Much	360 (38.5)
	General	246 (26.3)
	Little	28 (3.0)
	Stress-free	15 (1.6)
How are you going to handle this pressure?	Active preparation	547 (58.4)
	Continue education	282 (30.1)
	Lowering employment standards	33 (3.5)
	Negative waiting for	19 (2.0)
	Other	55 (5.9)
Will you continue to advise your students or children to study medicine?	Will take the initiative to suggest that teachers, younger siblings or children study medicine	187 (20.0)
	Will not take the initiative to suggest, and if there are teachers and younger sisters to consult, I will objectively explain the advantages and disadvantages of studying medicine	504 (53.8)
	Not recommended students to study medical related majors	91 (9.7)
	It is the best choice for students to choose their own major	112 (12.0)
	Other	42 (4.5)

Table 9 presented variables associated with changes in the employment situation before/after the pandemic, including major (*p* < 0.001), grade (*p* = 0.001), anxiety (*p* = 0.002), depression (*p* = 0.030), having considered career development after graduation (*p* = 0.039), and having clinical practice experience (*p* = 0.026). As seen from Table 10, level of education (*p* = 0.027), academic ranking (*p* < 0.001), stress (*p* = 0.007), anxiety (*p* < 0.001), depression (*p* < 0.001), insomnia (*p* < 0.001), having considered career development after graduation (*p* < 0.001), having attended any training or lectures about future career options (*p* < 0.001), employment region (*p* < 0.001), and expected employment income (*p* < 0.029) were linked with satisfaction with major before/after the pandemic. Medical students with junior college, ranking in the top 50% of students, without psychological health concerns, having considered career development after graduation, and having attended any training or lectures about their future career path were more satisfied with their majors than they were before the pandemic. Table 11 showed that medical students in China's western universities (*p* < 0.001), in nursing and other majors (*p* < 0.001), having attended junior college (*p* < 0.001), with average monthly household income < 5,000 yuan (*p* = 0.027), with depression (*p* = 0.048), having considered career development after graduation (*p* = 0.043), having attended any training or lectures about future career path (*p* < 0.001), and clinical practice experience were likely to change their career plans after going through this pandemic (*p* < 0.001). Additionally, medical students in China's western universities (*p* = 0.001), nursing students (*p* = 0.005), those with household incomes affected by the pandemic (*p* < 0.001), having considered career development after graduation (*p* = 0.031), having attended any training or lectures about their future career path (*p* = 0.018), having clinical practice experience (*p* = 0.015), and future employment regions in first-tier cities, hometowns or locations of their universities (*p* = 0.011) may create more pressure during employment after experiencing this outbreak (Table 12).

**Table 9:** Analysis of the factors affecting employment situation after the pandemic.

**General information**	**Variable**	**Classification**	**What do you think of the current employment situation after the pandemic?**			***p* value**
			**Worse than before**	**Almost**	**More opportunities than before**	
	Major	Nursing	181 (37.6)	201 (41.7)	100 (20.7)	0.000
		Clinical medicine	86 (42.8)	75 (37.3)	40 (19.9)	
		Others	104 (41.1)	69 (27.3)	80 (31.6)	
	Grade	Freshman	220 (46.8)	89 (18.9)	161 (34.3)	0.001
		Sophomore	177 (54.8)	83 (25.7)	63 (19.5)	
		Junior	56 (57.7)	19 (19.6)	22 (22.7)	
		Senior and intern	23 (62.2)	8 (21.6)	6 (16.2)	
		Master/PhD graduate student	6 (66.7)	2 (22.2)	1 (11.1)	
Psychological factors	Anxiety	Normal	364 (52.4)	131 (18.8)	200 (28.8)	0.002
		Symptom	118 (49.0)	70 (29.0)	53 (22.0)	
	Depression	Normal	352 (51.1)	137 (19.9)	200 (29.0)	0.030
		Symptom	130 (52.6)	64 (25.9)	53 (21.5)	
Employment intension	Have you thought about what you will do after graduation?	Yes	420 (52.4)	161 (20.1)	221 (27.6)	0.039
		No	62 (46.3)	40 (29.9)	32 (23.9)	
	Do you have clinical practice experience?	Yes	97 (58.4)	38 (22.9)	31 (18.7)	0.026
		No	385 (50.0)	163 (21.2)	222 (28.8)	

**Table 10:** Analysis of the factors affecting the professional satisfaction after the pandemic.

	**Variable**	**Classification**	**Are you more satisfied with your major after the pandemic?**			***p* value**
			**More satisfactory**	**Not much difference**	**Unsatisfactory**	
General information	Degree of education	Junior college	62 (42.2)	77 (52.4)	8 (5.4)	0.027
		Undergraduate	220 (30.6)	464 (64.6)	34 (4.7)	
		Graduate	18 (25.4)	47 (66.1)	6 (8.5)	
	Score ranking	Top 25%	93 (38.3)	138 (56.8)	12 (4.9)	0.000
		25%−50%	122 (35.0)	311 (60.5)	16 (4.6)	
		50%−75%	61 (24.2)	185 (73.4)	6 (2.4)	
		Below 75%	24 (26.1)	54 (58.7)	14 (15.2)	
Psychological factors	Stress	Normal	266 (32.5)	518 (63.2)	35 (4.3)	0.007
		Symptom	34 (29.1)	70 (59.8)	13 (11.1)	
	Anxiety	Normal	244 (35.1)	426 (61.3)	25 (3.6)	0.000
		Symptom	56 (23.2)	162 (67.2)	23 (9.5)	
	Depression	Normal	252 (36.6)	411 (59.7)	26 (3.8)	0.000
		Symptom	48 (19.4)	177 (71.7)	22 (8.9)	
	Insomnia	Normal	248 (36.7)	400 (59.3)	27 (4.0)	0.000
		Symptom	52 (19.9)	188 (72.0)	21 (8.0)	
Employment intension	Have you thought about what you will do after graduation?	Yes	277 (34.5)	489 (61.0)	36 (4.5)	0.000
		No	23 (17.2)	99 (73.9)	12 (9.0)	
	Have you attended any training or lectures related to your future career orientation?	Yes	147 (39.6)	210 (56.6)	14 (3.8)	0.000
		No	153 (27.1)	378 (66.9)	34 (6.0)	
	Employment region	First-tier city	150 (39.0)	215 (55.8)	20 (5.2)	0.000
		Where the student or school is located	137 (29.5)	305 (65.7)	22 (4.7)	
		Other	13 (14.9)	68 (78.2)	6 (6.9)	
	Expected employment income	3,000–8,000 yuan	138 (33.5)	258 (62.6)	16 (3.9)	0.029
		8,000–15,000 yuan	109 (32.0)	218 (63.9)	14 (4.1)	
		>15,000 yuan	53 (29.0)	112 (61.2)	18 (9.8)	

**Table 11:** Analysis of factors affecting career planning after the pandemic.

	**Variable**	**Classification**	**Has your career plan changed during this pandemic?**		***p* value**
			**Yes**	**No**	
General information	Location of universities	Eastern	86 (16.5)	436 (83.5)	0.000
		Western	108 (26.1)	306 (73.9)	
	Major	Nursing	95 (25.6)	276 (74.4)	0.000
		Clinical medicine	41 (11.9)	304 (88.1)	
		Others	58 (26.4)	162 (73.6)	
	Degree of education	Junior college	49 (33.3)	98 (66.7)	0.000
		Undergraduate	139 (19.4)	579 (80.6)	
		Graduate	6 (8.5)	65 (91.5)	
	Average monthly household income	< 5,000	100 (24.8)	303 (75.2)	0.027
		5,000–10,000	67 (17.7)	311 (82.3)	
		>10,000	27 (17.4)	128 (82.6)	
Psychological factors	Depression	Normal	132 (19.2)	557 (80.8)	0.048
		Symptom	62 (25.1)	185 (74.9)	
Employment intension	Have you thought about what you will do after graduation?	Yes	175 (21.8)	627 (78.2)	0.043
		No	19 (14.2)	115 (85.8)	
	Have you attended any training or lectures related to your future career orientation?	Yes	108 (29.1)	263 (70.9)	0.000
		No	86 (15.2)	479 (84.8)	
	Do you have clinical practice experience?	Yes	55 (33.1)	111 (66.9)	0.000
		No	139 (18.1)	631 (81.9)	

**Table 12:** Analysis of factors affecting employment pressure after the pandemic.

	**Variable**	**Classification**	**Do you feel the pressure of employment in the normalization context of the pandemic?**			***p* value**
			**Great/ larger**	**General**	**Not much/no pressure**	
General information	Location of universities	Eastern	334 (64.0)	162 (31.0)	26 (5.0)	0.001
		Western	313 (75.6)	84 (20.3)	17 (4.1)	
	Major	Nursing	282 (76.0)	74 (19.9)	15 (4.0)	0.005
		Clinical medicine	223 (64.6)	103 (29.9)	19 (5.5)	
		Others	142 (64.5)	69 (31.4)	9 (4.1)	
Knowledge about the pandemic	Have household incomes been affected by the pandemic?	Yes	457 (72.7)	153 (24.3)	19 (3.0)	0.000
		No	190 (61.9)	93 (30.3)	24 (7.8)	
Employment intension	Have you thought about what you will do after graduation?	Yes	565 (70.4)	205 (25.6)	32 (4.0)	0.031
		No	82 (61.2)	41 (30.6)	11 (8.2)	
	Have you attended any training or lectures related to your future career orientation?	Yes	270 (72.8)	92 (24.8)	9 (2.4)	0.018
		No	377 (66.7)	154 (27.3)	34 (6.0)	
	Do you have clinical practice experience?	Yes	127 (76.5)	29 (17.5)	10 (6.0)	0.015
		No	520 (67.5)	217 (28.2)	33 (4.3)	
	Employment region	First-tier city	267 (69.4)	102 (26.5)	16 (4.2)	0.011
		Where the student or school is located	330 (71.1)	117 (25.2)	17 (3.7)	
		Other	50 (57.5)	27 (31.0)	10 (11.5)	

### Relationship of variables with frontline work awareness against the COVID-19

Table 13 showed attitudes of medical students on the frontline work against the epidemic. After experiencing the epidemic, 60% of medical students thought that working in respiratory and infectious departments was more riskier, however, approximately 90% of medical students admired the work on the front lines against the pandemic and would like to be involved if they had the opportunity. Additionally, medical students in China's western universities (*p* < 0.001), female medical students (*p* < 0.001), those in nursing and other majors (*p* < 0.001), those with household incomes affected by the pandemic (*p* = 0.002), having clinical practice experience (*p* = 0.045), future employment in first-tier cities, hometowns or locations of the universities (*p* < 0.001) were inclined to think that risk of working in the respiratory and infectious disease departments was higher due to this outbreak (Table 14). Those with good psychological health (all *p* ≤ 0.001), no friends diagnosed with COVID-19 (*p* = 0.038), future employment in first-tier cities, hometowns or locations of the universities (*p* = 0.008) admired the work on the front lines against the pandemic and would have more liked to participate if they had the opportunity (Table 15).

**Table 13:** The awareness of frontline work against the COVID-19.

**Item**	**Statistical index**	**Population (*n*/%)**
After experiencing this epidemic, your understanding of the work risk of respiratory department and infectious department	The risk is higher in this department	563 (60.1)
	Perception not changed	345 (36.9)
	Risk of working this department is lower	28 (3.0)
Opinion on working on the front lines of the pandemic	Admire and would like to join if having the opportunity	831 (88.8)
	A little hesitant and worried about self-security	87 (9.3)
	Unwilling to participate	18 (1.9)

**Table 14:** Analysis of factors affecting risk cognition of working in respiratory department and infectious department.

	**Variable**	**Classification**	**After experiencing this epidemic, your understanding of the work risk in respiratory department and infectious department?**			***p* value**
			**Higher risk**	**Not changed**	**Lower risk**	
General information	Location of universities	Eastern	305 (58.4)	211 (40.4)	6 (1.1)	0.000
		Western	258 (62.3)	134 (32.4)	22 (5.3)	
	Gender	Male	134 (46.9)	143 (50.0)	9 (3.1)	0.000
		Female	429 (66.0)	202 (31.1)	19 (2.9)	
	Major	Nursing	229 (61.7)	121 (32.6)	21 (5.7)	0.000
		Clinical medicine	180 (52.2)	163 (47.2)	2 (0.6)	
		Others	154 (70.0)	61 (27.7)	5 (2.3)	
	Degree of education	Junior college	92 (62.6)	45 (30.6)	10 (6.8)	0.026
		Undergraduate	430 (59.9)	270 (37.6)	18 (2.5)	
		Graduate	41 (57.7)	30 (42.3)	0 (0.0)	
Knowledge about the pandemic	Have household incomes been affected by the pandemic?	Yes	402 (63.9)	207 (32.9)	20 (3.2)	0.002
		No	161 (52.4)	138 (45.0)	8 (2.6)	
Employment intension	Do you have clinical practice experience?	Yes	109 (65.7)	49 (29.5)	8 (4.8)	0.045
		No	454 (59.0)	296 (38.4)	20 (2.6)	
	Employment region	First-tier city	222 (57.7)	150 (39.0)	13 (3.4)	0.000
		Where the student or school is located	300 (64.7)	157 (33.8)	7 (1.5)	
		Other	41 (47.1)	38 (43.7)	8 (9.2)	

**Table 15:** Analysis of factors affecting the attitudes of frontline work against COVID-19.

	**Variable**	**Classification**	**Attitudes of frontline work against COVID-19**			***p* value**
			**Admire and would like to join if having the opportunity**	**A little hesitant and worried about self-security**	**Unwilling to participate**	
General information	Gender	Male	241 (84.3)	38 (13.3)	7 (2.4)	0.014
		Female	590 (90.8)	49 (7.5)	11 (1.7)	
	Home place	Metropolis	69 (82.1)	13 (15.5)	2 (2.4)	0.042
		Small-medium cities	403 (87.2)	50 (10.8)	9 (1.9)	
		Countryside	359 (92.1)	24 (6.2)	7 (1.8)	
Psychological factors	Stress	Normal	735 (89.7)	72 (8.8)	12 (1.5)	0.008
		Symptom	96 (82.1)	15 (12.8)	6 (5.1)	
	Anxiety	Normal	637 (91.7)	49 (7.1)	9 (1.3)	0.000
		Symptom	194 (80.5)	38 (15.8)	9 (3.7)	
	Depression	Normal	628 (91.1)	52 (7.5)	9 (1.3)	0.000
		Symptom	203 (82.2)	35 (14.2)	9 (3.6)	
Knowledge about the pandemic	Have you participated in epidemic prevention and control work	Yes	439 (91.3)	40 (8.3)	2 (0.4)	0.001
		No	392 (86.2)	47 (10.3)	16 (3.5)	
	Having family or friends infected with COVID-19 or not?	Yes	14 (77.8)	2 (11.1)	2 (11.1)	0.038
		No	817 (89.0)	85 (9.3)	16 (1.7)	
Employment Intension	Have you thought about what you will do after graduation?	Yes	721 (89.9)	71 (8.9)	10 (1.2)	0.000
		No	110 (82.1)	16 (11.9)	8 (6.0)	
	Do you have clinical practice experience?	Yes	152 (91.6)	8 (4.8)	6 (3.6)	0.023
		No	679 (88.2)	79 (10.3)	12 (1.6)	
	Employment region	First-tier city	343 (89.1)	36 (9.4)	6 (1.6)	0.008
		Where the student or school is located	420 (90.5)	37 (8.0)	7 (1.5)	
		Other	68 (78.2)	14 (16.1)	5 (5.7)	
	Expected employment income	3,000–8,000 yuan	372 (90.3)	32 (7.8)	8 (1.9)	0.003
		8,000–15,000 yuan	306 (89.7)	34 (10.0)	1 (0.3)	
		>15,000 yuan	153 (83.6)	21 (11.5)	9 (4.9)	

## Discussion

Our study showed differences in psychological problems of medical students in China's eastern and western universities in the post-epidemic era. The sources of psychological problems resulted from family economic and academic pressure. Improving awareness of epidemic prevention and control was beneficial in reducing psychological stress. Moreover, major, education level, academic ranking, family income, and clinical experience may affect the option of employment location and employment income. Impact of COVID-19 on household income may also affect the choice of employment location. Perception of epidemic prevention and control may change future employment income. Medical students with psychological problems thought that their employment situations became more intense due to the COVID-19 and were reluctant to actively make changes. Significantly, some active measures, such as conscientious consideration of employment, participation in career planning training lectures and timely adjustment of career planning, were helpful for professional identity of medical students.

Based on the results of our survey, there were differences about medical students' anxiety, career plan change and work pressure in China's eastern and western university. A Saudi Arabia survey observed nearly 20% of respondents suffering from a moderate to severe level of anxiety relative to this pandemic; western, northern, and eastern regions were reported to be the most anxious, which may be due to western and eastern regions being the initial epicenters of COVID-19 within Saudi Arabia (11). These findings are similar to those in Wuhan, China, the epicenter of COVID-19, with medical professionals experiencing a higher level of anxiety (12). These data demonstrate that differences in geographical area result in diversity in the degree of anxiety of medical students; however, the specific reasons still need to be explored in detail.

This study investigated the factors related to psychological abnormalities in medical students in the post-epidemic era. In concert with results from Shpakou et al.'s survey that medical students are less satisfied with life and have a higher level of perceived stress than other majors (13), our study found that nursing and clinical students were more likely to suffer from psychological stress, anxiety and insomnia. Of note, senior and graduate medical students reported less anxiety than younger students, and no close family member or friend infected with COVID-19 reduced medical students' risk of anxiety and depression, which was consistent with published reports. For instance, from a US survey, stress in medical students during COVID-19 is higher than it in pre-pandemic (14). Stress and burnout in second-/third-year students are at the highest levels, and tight finances and having themselves or family members diagnosed with COVID-19 causes higher stress (14). Having moderate or higher household economic levels is documented to be a preventive factor for improving students' psychological health problems (15), which echoes with our data showing that family income level caused anxiety. Evidence from Russian university students has demonstrated that students with COVID-19 experience are more likely to suffer from psychological anxiety, stress and somatic burden than those without COVID-19 experience (16), which is in accordance with this study revealing that acquaintances diagnosed with COVID-19 can increase medical students' psychological stress and even lead to insomnia. Similarly, another survey from Nigeria Medical University illuminates that a family income < 100,000 naira and having a relative/acquaintance infected with COVID-19 are independently relevant to psychological distress (17). Cipta et al.'s survey in Indonesia has illustrated that 35.5% of medical students experience burnout during the COVID-19 pandemic (18). Our data, combined with published findings, underline the importance of determining the related factors affecting psychological health of medical students to improve their psychological health.

Due to the impact of the COVID-19 pandemic, limited geographical mobility and intensive quarantine work in large cities were likely to affect the employment choices of medical students in the post-pandemic era (8). According to this survey, major, grade, family monthly income and family income led to differences in employment location. Major, education level, academic ranking, family monthly income, clinical experience, participation in epidemic prevention and control work, and care about COVID-19 infection influenced the options to increase employment income. Liu et al.'s survey has discovered that married female PhD students who are from urban areas and have a high household income are inclined to choosing first-tier city as employment, and from their simulation results, increasing monthly income causes raise of the probability of choosing the job in the third-tier city (19). These findings may provide available information with advancing attraction for government policy-maker.

Furthermore, in this work, medical students with psychological health problems, especially those with depression, believed that the epidemic led to a more difficult employment situation, lower satisfaction with their major, and were more inclined to change their career plans. Analogously, a survey with masters from all Polish medical schools reveals that 17% of respondents are willing to work at the pandemic frontline; ~40% of them declares the need for psychological or psychiatric consultation to address pandemic challenges, and some of them announces that their zeal toward the medical profession is affected by an increase in social aversion and mistrust toward doctors (20). In addition, Hagiya et al. discovers a very low proportion of interest in becoming an infectious disease specialist among medical students, which indicates that experience of COVID-19 discourages Japanese medical students from considering infectious disease as an option for their future careers (21). Particularly, a survey of medical students in Hubei Province demonstrates that grade level, attitude toward health care, and the level of the pandemic's impact on their lives significantly influence career intentions alterations (5). Previous studies demonstrate medical students' attitude toward COVID-19 and influence of it can affect career decisions. For example, fear of COVID-19 and life satisfaction are reported to be positive/negative effects on occupational turnover intention among nursing students (22). A Taiwan's study clarifies that prelicensure nursing students' attitudes toward the pandemic exert a direct impact on their perceived control and decision-making attitude about their career (23). The aforementioned data emphasize that determining associated factors influencing future employment decisions is crucial for formulating guidance strategies applicable to modifying medical students' careers.

Considering post-graduation options and attending training/lectures about future career prospects since entering university were conducive to reducing employment pressure caused by the epidemic, timely adjustments in career planning and increasing future career identity (24–26). From our survey, internship experience contributed to making adjustments based on career planning for medical students. Apart from it, there are other factors that produces an effect on career planning from previous studies as follow. A survey from Geneva suggests that enriched clinical exposure has a positive impact on relieving stress and enhancing professional identity (27). Another study of Chinese medical students points out that scant social support, depression, and higher grades are determined to negatively influence career attitude (28). Analogously, attitudes toward occupation, grades, major, and college choice are suggested to produce effects on the professional identity of medical students (25). Briefly, in the post-COVID-19 period, strengthening educational measures directed at career planning are warranted to strengthen medical students' professional identity.

This study also exists several limitations. First, the present study is based on cross-sectional survey that catches the influence of COVID-19 at a point in time and longitudinal influence is unclear. Second, introduction of data on non-medical students may be more powerful in illustrating the impact of COVID-19 on medical students. Third, the inclusion of multiple regions will be more meaningful to explore the role of regional differences in this study.

In summary, our results revealed multiple associated factors for medical students' psychological problems, future employment decision-making and professional identity. It is necessary to take effective measures, in accordance with local conditions, to intervene in the psychological problems of medical students that were triggered by the epidemic. Strengthening updated knowledge of COVID-19, actively guiding the employment planning of medical students according to individual needs, and strengthening career planning training and lectures will be conducive to promoting psychological health and positive career attitudes of medical students.

## Data availability statement

The original contributions presented in the study are included in the article/supplementary material, further inquiries can be directed to the corresponding author.

## Ethics statement

The studies involving human participants were reviewed and approved by Shandong First Medical University. The patients/participants provided their written informed consent to participate in this study. Written informed consent was obtained from the individual(s) for the publication of any potentially identifiable images or data included in this article.

## Author contributions

YW designed the study. JingxW, CY, JingzW, XS, and WS collected and analyzed all the data. YW and CY wrote the manuscript. All authors contributed to the article and approved the submitted version.

## References

[1] Lai J Ma S Wang Y Cai Z Hu J Wei N. Factors associated with mental health outcomes among health care workers exposed to coronavirus disease 2019. JAMA Netw Open. (2020) 3:e203976. 10.1001/jamanetworkopen.2020.3976 32202646 PMC7090843

[2] Zhou T Xu C Wang C Sha S Wang Z Zhou Y. Burnout and well-being of healthcare workers in the post-pandemic period of COVID-19: a perspective from the job demands-resources model. BMC Health Serv Res. (2022) 22:284. 10.1186/s12913-022-07608-z 35236354 PMC8888816

[3] Pinho R Costa TF. Mental health and burnout syndrome among postgraduate students in medical and multidisciplinary residencies during the COVID-19 pandemic in Brazil: Protocol for a prospective cohort study. JMIR Res Protoc. (2021) 10:e24298. 10.2196/24298 33290246 PMC7817252

[4] Zhang L Qi H Wang L Wang F Huang J Li F. Effects of the COVID-19 pandemic on acute stress disorder and career planning among healthcare students. Int J Ment Health Nurs. (2021) 30:907–16. 10.1111/inm.12839 34002465 PMC8242478

[5] Wang X-L Liu M-X Peng S Yang L Lu C Shou S-C. Impact of the COVID-19 pandemic on career intention amongst undergraduate medical students: a single-centre cross-sectional study conducted in Hubei Province. BMC Med Educ. (2022) 22:154. 10.1186/s12909-022-03201-4 35255878 PMC8901388

[6] Xie J Li X Luo H He L Guo Y. Depressive symptoms, sleep quality and diet during the 2019 novel Coronavirus epidemic in China: a survey of medical students. Front Public Health. (2020) 8:588578. 10.3389/fpubh.2020.588578 33575239 PMC7870982

[7] Liu J Zhu Q Fan W Makamure J Zheng C Wang J. Online mental health survey in a medical college in China during the COVID-19 outbreak. Front Psychiatry. (2020) 11:e459. 10.3389/fpsyt.2020.00459 32574242 PMC7237734

[8] Gong Z Li W Bu H He M Hou H Ma T. Impact of COVID-19 pandemic on the professional intention of medical and related students. BMC Med Educ. (2021) 21:484. 10.1186/s12909-021-02922-2 34503514 PMC8428501

[9] Lovibond PF Lovibond SH. The structure of negative emotional states: comparison of the Depression Anxiety Stress Scales (DASS) with the Beck Depression and Anxiety Inventories. Behav Res Ther. (1995) 33:335–43. 10.1016/0005-7967(94)00075-U 7726811

[10] Bastien CH Vallières A Morin CM. Validation of the Insomnia Severity Index as an outcome measure for insomnia research. Sleep Med. (2001) 2:297–307. 10.1016/S1389-9457(00)00065-4 11438246

[11] Mashel AF Yaagoub AH Saad ASD. Anxiety levels amid the COVID-19 lockdown in Saudi Arabia. Int J Gen Med. (2021) 14:2161–71. 10.2147/IJGM.S312465 34103971 PMC8180301

[12] Zhang C Peng D. Individual perceived stress mediates psychological distress in medical workers during COVID-19 epidemic outbreak in Wuhan. Neuropsychiatric Dis Treatment. (2020) 16:2529–37. 10.2147/NDT.S266151 33149594 PMC7604251

[13] Shpakou A Naumau IA Krestyaninova TY Znatnova AV Lollini SV Surkov S. Physical activity, life satisfaction, stress perception and coping strategies of university students in Belarus during the COVID-19 pandemic. Int J Environ Res Public Health. (2022) 19:8629. 10.3390/ijerph19148629 35886479 PMC9317606

[14] Alkureishi ML Jaishankar D Dave S Tatineni S Zhu M Chretien KC. Impact of the early phase of the COVID-19 pandemic on medical student well-being: a multisite survey. J Gen Intern Med. (2022) 37:2156–64. 10.1007/s11606-022-07497-2 35710675 PMC9202979

[15] Wang C Yan S Jiang H Guo Y Gan Y Lv C. Socio-demographic characteristics, lifestyles, social support quality and mental health in college students: a cross-sectional study. BMC Public Health. (2022) 22:1583. 10.1186/s12889-022-14002-1 35987998 PMC9392273

[16] Zolotareva A Belousova S Danilova I Tseilikman V Lapshin M Sarapultseva L. Somatic and psychological distress among Russian university students during the COVID-19 pandemic. Int J Psychiatry Med. 2022:912174221123444. 10.1177/00912174221123444 35998088 PMC9403531

[17] Idowu OM Adaramola OG Aderounmu BS Olugbamigbe ID Dada OE Osifeso AC. A gender comparison of psychological distress among medical students in Nigeria during the Coronavirus pandemic: a cross-sectional survey. Afr Health Sci. (2022) 22:541–50. 10.4314/ahs.v22i1.63 36032445 PMC9382491

[18] Cipta DA Wijovi F Melisa L Lili R Marcella E Tancherla A. Burnout prevalence and degree among undergraduate medical students in Indonesia during 1 month of the COVID-19 pandemic: A cross-sectional descriptive survey. Int J Soc Psychiatry. (2022) 68:1232–7. 10.1177/00207640221116812 36047053

[19] Liu S Gu Y Yang Y Schroeder E. Chen Y. Tackling brain drain at Chinese CDCs: understanding job preferences of public health doctoral students using a discrete choice experiment survey. Human Resourc Health. (2022) 20:46. 10.1186/s12960-022-00743-y 35606873 PMC9125964

[20] Forycka J Pawłowicz-Szlarska E Burczyńska A Cegielska N Harendarz K Nowicki M. Polish medical students facing the pandemic—assessment of resilience, well-being and burnout in the COVID-19 era. PLoS ONE. (2022) 17:e0261652. 10.1371/journal.pone.0261652 35073318 PMC8786167

[21] Hagiya H Otsuka Y Tokumasu K Honda H Nishimura Y Obika M. Interest in Infectious Diseases specialty among Japanese medical students amidst the COVID-19 pandemic: a web-based, cross-sectional study. PLoS ONE. (2022) 17:e0267587. 10.1371/journal.pone.0267587 35446911 PMC9022859

[22] Lin Y Hu Z Danaee M Alias H Wong LP. The impact of the COVID-19 pandemic on future nursing career turnover intention among nursing students. Risk Manag Healthc Policy. (2021) 14:3605. 10.2147/RMHP.S322764 34475792 PMC8407786

[23] Lin S-C Ni L-F Wang Y-M Lee SH Liao H-C Huang C-Y. Prelicensure nursing students' COVID-19 attitude impact on nursing career decision during pandemic threat in Taiwan: a cross-sectional study. Int J Environ Res Public Health. (2021) 18:3272. 10.3390/ijerph18063272 33809956 PMC8004179

[24] Park GM Hong AJ. “Not yet a doctor”: medical student learning experiences and development of professional identity. BMC Med Educ. (2022) 22:146. 10.1186/s12909-022-03209-w 35246116 PMC8896319

[25] Lai T Liang W Zhong M Zhu P Li B. Current status of Chinese medical students' professional identity after COVID-19 and the factors that influence it. Front Psychol. (2022) 13:816767. 10.3389/fpsyg.2022.816767 35693496 PMC9174680

[26] Fris DAH van Vianen AEM Koen J de Hoog M de Pagter APJ. Medical students' career decision-making stress during clinical clerkships. Perspect Med Educ. (2022) 11:350–8. 10.1007/s40037-022-00734-8 36478525 PMC9734734

[27] Wurth S Sader J Cerutti B Broers B Bajwa NM Carballo S. Medical students' perceptions and coping strategies during the first wave of the COVID-19 pandemic: studies, clinical implication, and professional identity. BMC Med Educ. (2021) 21:1–11. 10.1186/s12909-021-03053-4 34915888 PMC8674407

[28] Yang X Gao L Zhang S Zhang L Zhang L Zhou S. The professional identity and career attitude of Chinese medical students during the COVID-19 pandemic: a cross-sectional survey in China. Front Psychiatry. (2022) 13:774467. 10.3389/fpsyt.2022.774467 35242061 PMC8886109

